# Sleep Promotes the Extraction of Grammatical Rules

**DOI:** 10.1371/journal.pone.0065046

**Published:** 2013-06-05

**Authors:** Ingrid L. C. Nieuwenhuis, Vasiliki Folia, Christian Forkstam, Ole Jensen, Karl Magnus Petersson

**Affiliations:** 1 Donders Institute for Brain, Cognition, and Behaviour, Radboud University Nijmegen, Nijmegen, The Netherlands; 2 Sleep and Neuroimaging Laboratory, Department of Psychology, University of California, Berkeley, California, United States of America; 3 Max Planck Institute for Psycholinguistics, Nijmegen, The Netherlands; 4 Cognitive Neurophysiology Research Group, Stockholm Brain Institute, Karolinska Institutet, Department of Clinical Neuroscience, Stockholm, Sweden; 5 Cognitive Neuroscience Research Group, Institute of Biotechnology & Bioengineering, Centre for Molecular and Structural Biomedicine, Universidade do Algarve, Campus de Gambelas, Faro, Portugal; Harvard Medical School, United States of America

## Abstract

Grammar acquisition is a high level cognitive function that requires the extraction of complex rules. While it has been proposed that offline time might benefit this type of rule extraction, this remains to be tested. Here, we addressed this question using an artificial grammar learning paradigm. During a short-term memory cover task, eighty-one human participants were exposed to letter sequences generated according to an unknown artificial grammar. Following a time delay of 15 min, 12 h (wake or sleep) or 24 h, participants classified novel test sequences as Grammatical or Non-Grammatical. Previous behavioral and functional neuroimaging work has shown that classification can be guided by two distinct underlying processes: (1) the holistic abstraction of the underlying grammar rules and (2) the detection of sequence chunks that appear at varying frequencies during exposure. Here, we show that classification performance improved after sleep. Moreover, this improvement was due to an enhancement of rule abstraction, while the effect of chunk frequency was unaltered by sleep. These findings suggest that sleep plays a critical role in extracting complex structure from separate but related items during integrative memory processing. Our findings stress the importance of alternating periods of learning with sleep in settings in which complex information must be acquired.

## Introduction

Considerable evidence suggests that new skill learning or acquisition of new declarative information does not only take place during training or exposure to new material, but continues to develop during offline periods following learning in which one is not directly engaged in the task. Both offline time spent awake and asleep can be beneficial [1], [2], [3]. Recently, studies have indicated that offline time might promote the formation of relations between pieces of information. For example, in transitive inference learning, the association of related memories preferentially develops following offline time periods, with the greatest improvement after sleep [4]. Also, following exposure to an artificial language, infants learn simple relations between syllables only after a period of sleep [5]. It has therefore been hypothesized that sleep is specifically beneficial for generalization and abstraction [6].

A grammar specifies a complex set of relations between words that supports us in comprehending how concepts are interrelated in sentences, for example, in determining who did what to whom. Acquiring a new grammar requires complex rule extraction. The human brain is equipped with implicit learning mechanisms that extract such patterns from the information to which it is exposed [7], [8] (note, we use the terms “implicit” and “implicit learning” in their classical sense, meaning a lack of meta-cognitive knowledge and in particular the absence of any stated use of explicit “problem solving” strategies [7], [8], [9]). While above findings have begun to establish a role for sleep in associative memory processing, it is currently unknown whether offline (sleep) time contributes to the implicit abstraction of complex structure needed for grammar acquisition. Moreover, the mechanistic route(s) by which these offline benefits develop remain largely uncharacterized.

**Figure 1:**
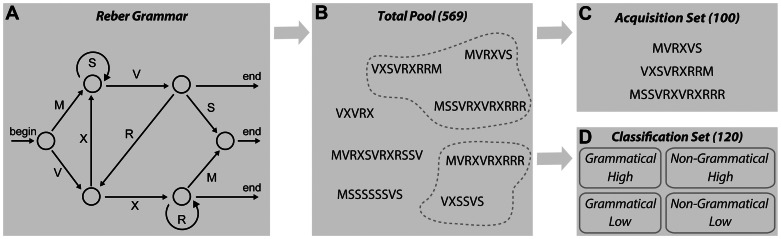
Artificial grammar and stimulus material. A) The Reber grammar used. B) Total Pool: all 569 unique 5–12 letter sequences. C) Acquisition Set: 100 representative sequences, used in the short-term memory task. D) Classification Set: 120 sequences arranged in a 2×2 factorial design, with the factors grammaticality (Grammatical, Non-Grammatical), and associative chunk strength (High, Low), used in the classification task.

Artificial grammar learning has been extensively used to study grammar acquisition (e.g. [7], [8], [9], [10], [11], [12], [13], [14]). In such tasks, a complex set of rules constituting the grammar (see for instance **Fig. 1A**), defines the order of symbols in a sequence. The typical artificial grammar learning experiment includes a short acquisition session followed by a classification test. During the acquisition phase, participants are engaged in a short-term memory task using an acquisition sample generated from a grammar. After the acquisition session, the participants are informed that the sequences were generated according to a complex system of rules, however, no information about the rules is provided. The participants then have to classify new items as Grammatical or Non-Grammatical guided by their immediate intuitive impression (i.e., guessing based on “gut feeling”). The participants usually perform reliably above chance, suggesting that they acquired knowledge about relevant aspects of the underlying grammar [13], [14]. Based on the fact that subjects are typically unable to provide sufficient, if any, reasons to motivate their classification decisions it is assumed that the classification performance is based largely on implicit acquisition mechanisms, for reviews see [9], [11], [15], [16] however also see [17], [18], [19]. It has been suggested that artificial grammar learning depends on the implicit acquisition of structural knowledge (i.e., “rule-based” representations). This knowledge could be extracted from the combined sample of acquisition sequences by integrative processes combining the structural information present in the individual sequences [7], [8], [12].

Alternative theoretical frameworks have questioned the acquisition of abstract (“rule”) knowledge during artificial grammar learning and suggest instead that what participants actually learn are parts of the presented sequences (“chunks” such as bigrams and trigrams). The acquired knowledge of these chunks would subsequently be sufficient to account for the performance of grammaticality judgments [20], [21]. In order to address this issue the associative chunk strength (ACS) measure was developed [11], [12]. ACS is quantified in terms of the frequency with which local subsequences (e.g., bi- and trigrams) of a classification item occurred in the Acquisition Set. In this approach, acquired structural (“rule based”) and instance specific information are quantified by grammaticality status and ACS, respectively. Several studies have been performed to test the independent contributions of grammaticality and ACS on classification by arranging the sequences in the Classification Set in a 2×2 factorial design (e.g. [11], [12], [14], [22], [23], [24]). In these studies the Classification Set contains both items that violate the grammar but include many highly frequent subsequences, and Grammatical sequences that omit highly frequent subsequences. From the studies in which the ACS of the test items was manipulated, it is clear that both grammaticality and ACS affect grammar judgments made by the participants. However, when participants perform robustly (e.g., after several days of implicit acquisition) the effects are largely independent and the grammaticality effect is significantly larger than the effect of ACS [13], [14], [25]. In addition, functional neuroimaging data show that the effects of grammaticality and ACS affect different brain regions and typically no interaction between these factors are found in the brain [22], [26].

Taken together, the evidence suggests that artificial grammar learning can be conceptualized both in terms of structure-based rule acquisition and surface-based statistical learning mechanisms. It is, however, unknown how an offline delay between acquisition and classification interacts with these mechanisms. Interestingly, many artificial grammar studies, especially those that investigate more extensive and complex artificial grammars, apply an acquisition phase that spans over several consecutive days (e.g. [13], [14], [22], [27]). This suggests that offline wake and/or sleep time between acquisition and classification might benefit artificial grammar learning.

Building on the emerging findings of a role for offline time in associative memory processing, the current study investigates whether offline wake and/or sleep time facilitates artificial grammar learning. Moreover, we specifically examine which mechanistic route (or combination of routes) mediates such delayed memory benefits by using ACS controlled classification items. Given that offline time might promote the formation of relations between pieces of information [4], [5] and the hypothesis that sleep is particularly beneficial for generalization and abstraction [6], we hypothesize that (1) offline time, and sleep in particular, will enhance artificial grammar learning; and that (2) offline (sleep) time specifically benefits abstraction of grammar rules without enhancing the effect of ACS.

## Materials and Methods

### Ethics Statement

This study was approved by the ethical committee (Comissie Mens Gebonden Onderzoek Regio Arnhem-Nijmegen). Subjects gave written informed consent to participate in this study.

### Participants

Eighty-one healthy participants participated in the study (66 female, age: mean 21.6, SD 2.41). Exclusion criteria included: traveling to another time zone within the past 3 months, current use of medication, as well as past or current neurological, psychiatric, or sleeping disorders. Participants were instructed to keep their normal sleep schedule, not to take any day time naps and refrain from alcohol from the night before to the end of the experiment. Compliance was verified using a questionnaire (self-report) at the beginning of the test phase. No participants reported day-time naps or drinking alcohol during the delay between acquisition and testing.

### Stimulus Material

The sequences were generated according to the Reber grammar [7] shown in **Figure 1A**. The sequences consisted of consonants from the alphabet {M, S, V, R, X}. A Grammatical sequence was generated by starting at “begin”, and following the arrows until an “end” is reached. For instance, from “begin”, to up-right (M), right (V), down-left (R), up (X), right (V), down-right (S), right (“end”), creates the sequence “MVRXVS”. All possible 5 to 12 letter sequences were generated, resulting in a Total Pool of 569 unique Grammatical sequences (**Fig. 1B**). The associative chunk strength (ACS) of all 2 and 3 letter chunks was calculated, i.e. the number of times each chunk appeared in the Total Pool.

The Acquisition Set (**Fig. 1C**), which was used to expose the participants to the grammar during the short-term memory task, contained 100 sequences that were selected from the Total Pool. The Acquisition Set was a representative sample of the grammar. Importantly, the chunks appeared in the same proportions in the Acquisition Set as they were present in the Total Pool (by selecting the best match over 1000 random draws of possible Acquisition Sets). The ACS of each sequence was calculated by averaging the associate strengths of each chunk in the sequence (**Table 1**). The ACS of the terminal chunks was calculated separately as well by isolating the bigram and trigram located at the beginning of the sequence and the bigram and trigram located at the end of the sequence and calculating their frequencies in the same locations in the sequences of the Total Pool (for more details see [11], [12]). The same Acquisition Set was used for all participants.

**Table 1:** Stimulus material.

	n	Mean ACS	% of stimulus type per sequence length			
			5–6 letters	7–8 letters	9–10 letters	11–12 letters
**Acquisition Set**	100	59.07 (7.8) [37.6–72.57]	2	16	30	52
**Classification Set**						
GH	30	59.93 (5.24) [50.09–71.29]	0	10	20	70
GL	30	40.77 (8.56) [20.46–48.82]	10	10	37	43
NGH	30	59.18 (5.36) [49.18–77.00]	0	10	20	70
NGL	30	40.94 (8.65) [20.46–49.82]	10	10	37	43

Standard deviations in parenthesis and range in brackets. ACS = associative chunk strength, G = Grammatical, NG = Non-Grammatical, H = High ACS, L = Low ACS.

The Classification Set, which was used during the classification test, contained 120 sequences. These sequences were arranged in a 2×2 factorial design, containing four sequence types of 30 sequences each: Grammatical & High ACS, Grammatical & Low ACS, Non-Grammatical & High ACS, and Non-Grammatical & Low ACS (**Fig. 1D**). The Classification Set was constructed by first selecting 60 sequences from the remaining Total Pool, of which 30 had a High ACS and 30 a Low ACS. Calculating the ACS was done using a similar procedure as described above, but this time chunk strength was determined in relation to the Acquisition Set and not the Total Pool. These 60 sequences formed the Grammatical sequences. Subsequently, the Non-Grammatical sequences were constructed by modifying these 60 Grammatical items: two (non-terminal) letters were replaced with another consonant of the {M, S, V, R, X} alphabet, in such a way that the resulting sequence could not be generated by the original grammar. For instance the third and fifth letter of the Grammatical sequence “VXSSVS”, could be altered into the Non-Grammatical sequence “VX***R***S***S***S”. Importantly, the ACS (both overall, and for terminal positions only) was kept equal between the Grammatical and Non-Grammatical sequences. This was accomplished by generating all possible Non-Grammatical sequences for each Grammatical sequence, and selecting the Non-Grammatical sequence that was most equal in ACS to the Grammatical sequence. The same Classification Set was used for all participants; see **Table 1** for details of ACS per stimulus category.

### Experimental Design

**Figure 2:**
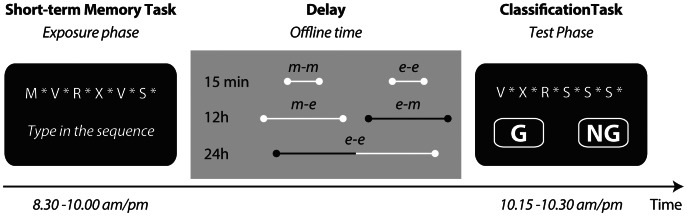
Procedure. Exposure to the grammar took place during a short-term memory task. Each sequence was centrally presented letter-by-letter on a computer screen (2.7–6.9 s corresponding to 5–12 letters; 300 ms letter presentation, * = 300 ms inter-letter interval). Participants were randomly assigned to one of five groups with offline delays of varying length (15 min, 12 h, and 24 h). The delay of two groups contained nocturnal sleep (black lines, 12 h e-m and 24 h e-e). During the test phase, participants judged if novel sequences were Grammatical (G), or Non-Grammatical (NG). The letters were presented one by one, similarly to the short-term memory cover task. m = morning, e = evening.

The experiment was divided into two sessions involving, an exposure phase, and a test phase, separated by an offline delay period (**Fig. 2**). The current study investigated how the delay period modulates the effect that the grammaticality and the ACS of the test items have on the grammaticality judgment during the test phase. The response variable in this analysis was the endorsement rate (i.e., proportion of sequences classified as Grammatical independent of the actual grammaticality status of the items). Thus, endorsed Grammatical sequences can be interpreted as ‘hits’ while endorsed Non-Grammatical sequences are ‘false alarms’. We hypothesized that sleep during the delay increases the effect of the grammaticality on classification. However the length of the offline delay and the time of the exposure and test phase (morning or evening) could also potentially affect performance during the test phase. Therefore, the experiment contained 5 independent groups of participants that differed in the brain state during the delay (wake or sleep), the length of the delay (15 min, 12 h or 24 h), and the time of the exposure and testing phases (morning or evening; see **Fig. 2B** middle panel, and **Table 2**). Unfortunately, it is not possible to independently manipulate the length of the delay, the brain state during delay, and the exposure and testing times (for instance, it is not possible to have a group with a 15 min delay, containing sleep, and with exposure in the morning and testing in the evening) to obtain a full factorial design.

**Table 2:** Experimental groups.

Group	n	Acquisition	Test	Lengthdelay	Brainstatedelay
15 min morning	17	morning	morning	15 min	wake
15 min evening	15	evening	evening	15 min	wake
12 h wake	16	morning	evening	12 h	wake
12 h sleep	16	evening	morning	12 h	sleep
24 h sleep	17	evening	evening	24 h	sleep

Short-term memory performance during the exposure phase was quantified as the percentage of letters correct over all sequences used in the short-term memory task. We counted the number of correct letters from both the start of the sequence until an error was made, and from the end back until an error was made. The number of correct letters was the maximum of the two. For instance, if the sequence was VXSVRXRRM and the participant typed VXSVRRM the amount of correct letters was five, and if the participant typed MXSVRRM, the amount correct was three.

### Statistical Analysis

First, we performed repeated measures ANOVAs within each of the 5 independent groups of subjects separately (see **Table 2** for groups) to investigate the effect of grammaticality (Grammatical, Non-Grammatical) and ACS (High, Low) on endorsement. To quantify the ability to discriminate between Grammatical and Non-Grammatical sequences we also calculated d-prime, which provides the separation between the means of the signal (endorsed Grammatical items) and the noise (endorsed Non-Grammatical items) distributions in units of the standard deviation of the noise distribution (z-transformation).

To optimally investigate the effect of brain state, length of the delay, and time of the exposure and testing we performed a mixed model 3-Way factorial ANOVA with planned comparisons. This model contained 2 within subject factors: grammaticality (with the levels Grammatical and Non-Grammatical) and ACS (with the levels High and Low), and one between subject factor: condition (with the levels 15 min morning, 15 min evening, 12 h wake, 12 h sleep and 24 h sleep). With the four planned orthogonal contrasts we investigated the effects of brain state (wake, sleep, contrast 1) length of the delay (15 min, 12 h, contrast 2; 12 h, 24 h, contrast 3), and time of the exposure and testing (morning, evening, contrast 4; see **Table 3**). Since all contrasts were independent, no correction for multiple comparisons was necessary (e.g. [28]).

**Table 3:** Planned comparisons.

Contrast	Effect	15 min morning	15 min evening	12 h wake	12 h sleep	24 h sleep
1	Brain state	+2	+2	+2	−3	−3
2	Length of delay (a)	+1	+1	−2	0	0
3	Length of delay (b)	0	0	0	+1	−1
4	Time exposure, testing	+1	−1	0	0	0

Four orthogonal planned contrasts were tested to investigate the effects of brain state, length of delay and time of exposure and testing.

To follow up on effects found in the contrast testing for the effect of brain state, we performed post hoc T-tests, using a Bonferroni correction for multiple comparisons. To compare performance on the short-term memory task (acquisition phase) we performed a 1-Way factorial ANOVA with the same planned comparisons (see **Table 3**). This model contained the factor condition (with 5 levels) as between subject factor. All statistical analyses were performed using PASW/SPSS software with default settings unless stated otherwise. The default Type-III sum of squares was used to prevent confounding due to unequal group-sizes. P-values below 0.05 were considered significant.

### Procedure

The purpose of the short-term memory task (exposure phase) was to expose the participants to a large amount of sequences that all followed the Reber grammar. Participants were not informed that an underlying grammar was present (see **Appendix S1A** and **Appendix S1B**, for task instructions). During the short-term memory task (**Fig. 2**, left) one hundred sequences were presented twice, each time in random order. The five to twelve letters in each sequence were presented sequentially on a computer screen (2.7–6.9 s corresponding to 5–12 letters; 300 ms letter presentation, 300 ms inter-letter interval during which a * was presented). Each time, after the last letter of the sequence had disappeared from screen, the participants typed the whole sequence from memory on a keyboard. They did not receive any performance feedback. The short-term memory task always took place at 8∶30 (morning or evening), and lasted approximately 1.5 h, with a short break halfway through the task. During the delay (**Fig. 2**, middle), which followed the short-term memory task, the participants of the 12 h and 24 h groups left the laboratory to continue their standard daily activities and normal nocturnal sleep (sleep groups).

The classification session, which followed the delay, started with informing the participants that the sequences used in the short-term memory task were generated according to a complex set of rules (see **Appendix S2A** and **Appendix S2B** for task instructions). During the classification task (**Fig. 2**, right), novel sequences were presented, again letter-by-letter (same presentation rate as during the short-term memory task), after which the participants had to classify the sequences as Grammatical or Non-Grammatical. The participants were instructed to judge the sequence as a whole, and to respond immediately according to their intuitive impression or guess, based on ‘gut-feeling’. The classification task contained 120 sequences, presented once, in random order. The classification task testing phase started at 10∶15 (morning or evening).

## Results

### The Effect of Grammaticality and ACS on Endorsement within Each Group

**Figure 3:**
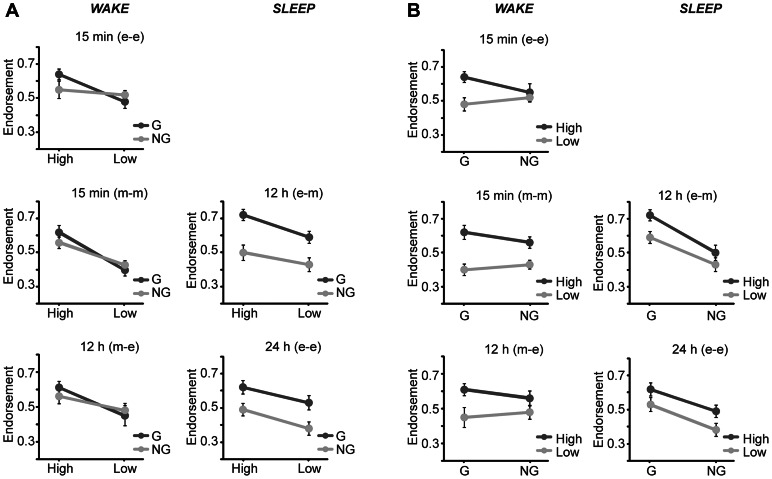
Endorsement for the four categories of test items within each of the 5 participant groups. A) In the three wake groups (left column), Grammatical (G) and Non-Grammatical (NG) items were endorsed with almost equal rates. In the sleep groups (right column), however, Grammatical test sequences were clearly endorsed more often than Non-Grammatical ones, both for items with High and Low ACS. B) In all groups, participants endorsed more sequences with a High than a Low ACS. Endorsement is the proportion of sequences classified as Grammatical independent of the actual grammaticality status of items. Error bars denote standard error of the mean.

**Table 4** and **Figure 3** show the endorsement rates during the classification task for the four stimulus types for each of the 5 groups. Within all 5 groups a significant main effect of ACS was present (all P<0.01), whereas only the sleep groups showed a main effect of grammaticality (sleep groups both P<0.013, wake groups, all P>0.59, **Table 5**). Thus, participants in all groups endorsed sequences with High ACS more often than sequences with Low ACS (**Table 4**, **Fig. 3B**), while only participants in the sleep groups endorsed significantly more Grammatical than Non-Grammatical sequences (**Table 4**, **Fig. 3A**). In addition, there was a significant interaction between grammaticality and ACS in the 15 min groups (both P = 0.02, **Table 5**). This suggests that participants might endorse more Grammatical than Non-Grammatical sequences with High ACS, however, this effect failed to reach significance in post hoc T-tests (15 min morning [T(16) = 1.6, P = 0.14], 15 min evening [T(14) = 1.4, P = 0.20]).

**Table 4:** Endorsement rates per group.

	Endorsement per stimulus category				Endorsement main factor level				D-prime
Group	GH	GL	NGH	NGL	G	NG	H	L	Z_G_–Z_NG_
15 min morning	0.62 (0.16)	0.40 (0.13)	0.56 (0.13)	0.43 (0.10)	0.51 (0.10)	0.49 (0.09)	0.59 (0.12)	0.41 (0.10)	0.02 (0.19)
15 min evening	0.64 (0.13)	0.48 (0.16)	0.55 (0.21)	0.52 (0.17)	0.55 (0.13)	0.53 (0.17)	0.59 (0.12)	0.50 (0.08)	0.06 (0.42)
12 h wake	0.61 (0.14)	0.45 (0.23)	0.56 (0.17)	0.48 (0.16)	0.52 (0.15)	0.52 (0.15)	0.59 (0.10)	0.46 (0.14)	0.01 (0.41)
12 h sleep	0.72 (0.13)	0.59 (0.14)	0.50 (0.18)	0.43 (0.16)	0.66 (0.12)	0.46 (0.15)	0.61 (0.11)	0.51 (0.09)	0.32 (0.39)
24 h sleep	0.62 (0.16)	0.53 (0.17)	0.49 (0.15)	0.38 (0.16)	0.59 (0.15)	0.43 (0.13)	0.55 (0.09)	0.46 (0.12)	0.24 (0.35)

Mean endorsement rates per stimulus category, on main factor level, and d-prime. Endorsement is the proportion of sequences classified as Grammatical independent of the actual grammaticality status of items. D-prime was defined as the difference between the z-transformed endorsement rate of Grammatical items and the z-transformed endorsement rate of the Non-Grammatical items. G = Grammatical, NG = Non-Grammatical, H = High ACS, L = Low ACS. Values in parenthesis denote standard deviations.

**Table 5:** Results of statistical tests within each group.

	ACS			Grammaticality			ACS×Grammaticality			D-prime		
Group	F	df	P	F	df	P	F	df	P	T	df	P
15 min morning	21	(1,16)	<0.001	0.28	(1,16)	0.60	6.2	(1,16)	0.02	0.48	16	0.64
15 min evening	9.3	(1,14)	0.009	0.15	(1,14)	0.71	6.9	(1,14)	0.02	0.56	14	0.59
12 h wake	9.7	(1,15)	0.007	0.006	(1,15)	0.94	2.4	(1,15)	0.14	0.12	15	0.91
12 h sleep	9.1	(1,15)	0.009	13	(1,15)	0.002	1.9	(1,15)	0.19	3.35	15	0.004
24 h sleep	10	(1,16)	0.006	8.1	(1,16)	0.012	0.27	(1,16)	0.61	2.76	16	0.014

Left: results of 2-way factorial (grammaticality and ACS) repeated measures ANOVA. Right: results of one sample t-test on d-prime values. Note: only the sleep groups showed a significant main effect of grammaticality and a d-prime value that was significantly higher than zero.

In line with the ANOVA results, the d-prime analysis showed that only the participants in the two sleep groups could significantly discriminate between Grammatical and Non-Grammatical sequences (sleep groups P<0.015, wake groups P>0.58, **Table 5**).

Thus, while the 5 independent groups differ with regard to brain state (wake, sleep), length of delay (15 min, 12 h and 24 h), and time of exposure (morning and evening) and testing (morning and evening), the classification behavior appears similar within the three wake groups (**Fig. 3A and 3B** left columns) and within the two sleep groups, respectively (**Fig. 3A and 3B** right columns).

### Differences between the Sleep and Wake Groups

We subsequently investigated how group membership (**Table 2**) affected the endorsement rates. In the mixed model ANOVA, which included the participants of all groups, we found main effects of grammaticality ([F(1, 76) = 11, P = 0.001]) and ACS ([F(1, 76) = 58, P<0.001]), and an interaction effect of grammaticality**×**ACS ([F(1, 76) = 6.5, P = 0.013]). More interestingly, when we looked at the interactions between the group the participants were part of and grammaticality and ACS, we found a significant group**×**grammaticality interaction (P = 0.032), while group**×**ACS (P = 0.43), and group**×**grammaticality**×**ACS (P = 0.20) were non-significant (**Table 6**, main level). Thus, in general both the grammaticality and the ACS of the test items affected the endorsement rate. However the degree to which ACS influenced classification was the same for all groups, while group membership did change the size of the effect of grammaticality.

**Table 6:** Results of 3-way mixed ANOVA with planned comparisons.

	ACS×group			Grammaticality×group			ACS×grammaticality×group		
	F/T	df	P	F/T	df	P	F/T	df	P
**Main level**	F = 0.97	(4,76)	0.43	F = 2.79	(4,76)	0.032	F = 1.56	(4,76)	0.20
**Contrast 1**	T = −1.01	(76)	0.32	T = 3.27	(76)	0.0016	T = 2.02	(76)	0.047
**Contrast 2**	T = −0.32	(76)	0.75	T = −0.24	(76)	0.81	T = 0.47	(76)	0.64
**Contrast 3**	T = 0.058	(76)	0.95	T = −0.66	(76)	0.51	T = 1.27	(76)	0.21
**Contrast 4**	T = −1.62	(76)	0.11	T = 0.13	(76)	0.90	T = −0.52	(76)	0.61

Results at main level reflect testing the null hypothesis of equal means between all 5 groups. Contrast 1 tested effect of brain state (wake, sleep), contrast 2 tested effect of length of delay within wake groups (12 h, 15 min), contrast 3 tested effect of length of delay within sleep groups (24 h, 12 h), and contrast 4 tested effect of time of day within the 15 min wake groups (morning, evening). See **Table 3** and Methods for more details.

**Figure 4:**
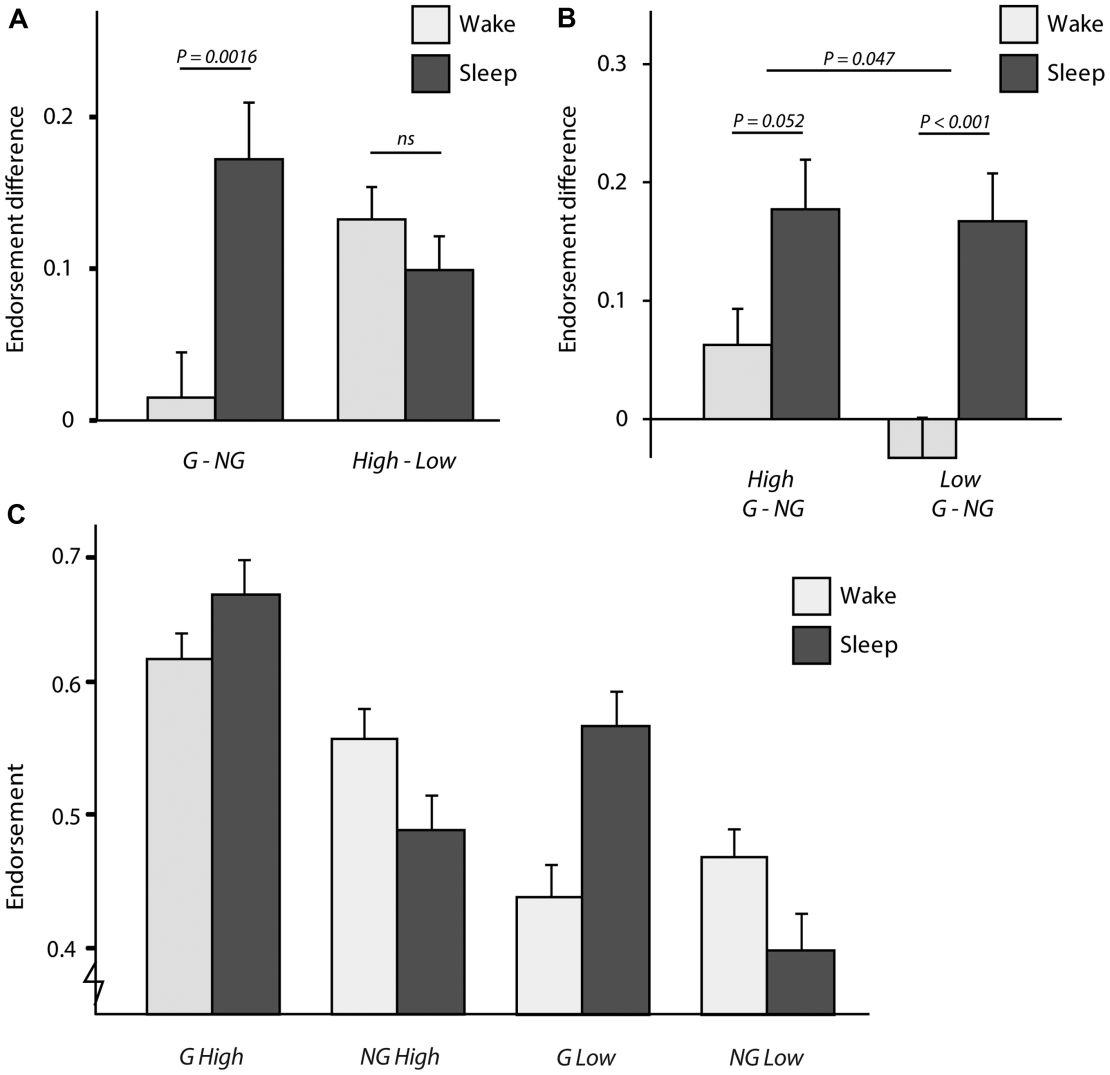
The effect of brain-state (wake, sleep) on classification performance. A) In the wake group (pooled 15 min morning, 15 min evening, and 12 h wake groups) participants endorsed almost equal amounts of Grammatical (G) and Non-Grammatical (NG) test items. In the sleep group (pooled 12 h sleep and 24 h sleep groups), however, the difference in the endorsement between G and NG items was more than 0.17. Thus, sleep increased the effect of grammaticality on endorsement (significant interaction between brain state and grammaticality in contrast 1, **Table 6**). Both in the wake and in the sleep group, participants endorsed more High than Low ACS items. However, sleep did not amplify that effect (no interaction between brain state and ACS in contrast 1, **Table 6**). B) Sleep especially enhanced the effect the grammaticality status of Low ACS test items had on classification (post hoc T-test, P<0.001, Bonferroni corrected), but also in the High ACS items there is a trend that sleep increased the effect of grammaticality (post hoc T-test, P = 0.052 Bonferroni corrected). Sleep increased the effect of the grammaticality on endorsement more for Low than High ACS items (brain state**×**grammaticality**×**ACS 3-Way interaction, P = 0.047 in contrast 1, **Table 6**). C) The endorsement of each of the four categories of sequences in the Classification Set (**Fig. 1D**) for the wake and sleep groups. Endorsement is the proportion of sequences classified as Grammatical independent of the actual grammaticality status. Endorsement difference is the difference between those proportions for G and NG sequences, or High ACS and Low ACS sequences. Error bars denote standard errors of the mean.

The first planned comparison (contrast 1, **Table 3**) tested whether the brain state (sleep or wake) during the offline delay influenced the effect of grammaticality and ACS on endorsement. We found a significant interaction effect between grammaticality and brain state (P = 0.0016) but no effect of brain state on ACS (P = 0.32; **Table 6**, contrast 1; **Fig.** **4A**). Moreover, the interaction between grammaticality, ACS, and brain state was significant (P = 0.047; **Fig. 4B**). The interaction effect between grammaticality and brain state suggests that sleep increased the effect of grammaticality on classification (the difference in endorsement between Grammatical and Non-Grammatical items increased; **Fig. 4A**), while it did not change the effect of ACS on classification (the difference in endorsement between High and Low ACS items did not change; **Fig. 4A**).

To interpret the 3-Way interaction between brain state, grammaticality, and ACS, the endorsement of each of the four types of test sequences was plotted separately per group (wake, sleep) in **Figure 4C**. Additionally, **Figure 4B** shows the effect of grammaticality for the High and Low ACS sequences separately. It is clear how especially the endorsement of the Low ACS sequences is influenced by sleep; the proportion of endorsed Low Grammatical items increases while the Low Non-Grammatical sequences are less often classified as Grammatical after sleep (**Fig. 4C**). The 3-Way interaction can be interpreted meaning that sleep increased the effect of grammaticality in Low ACS more than the effect of grammaticality in High ACS sequences. Post-hoc T-tests revealed that sleep very reliably increased the effect of grammaticality on endorsement in sequences with Low ACS (Grammatical Low - Non-Grammatical Low in pooled wake versus pooled sleep groups [T(79) = 3.77, P<0.001, Bonferroni corrected p-value]). But also in sequences with High ACS there was a trend that sleep increased the effect of grammaticality (Grammatical High - Non-Grammatical High in pooled wake versus pooled sleep groups [T(79) = 2.27, P = 0.052, Bonferroni corrected p-value]) however, to a lesser degree than in Low ACS test sequences, hence the significant 3-Way interaction.

Taken together, this suggests that before sleep, participants based their classification decisions on ACS, while they could hardly discriminate between Grammatical and Non-Grammatical items. However, after sleep, participants could reliably discriminate between Grammatical and Non-Grammatical test items. In particular, sleep enhanced the ability to classify sequences that did not contain many chunks that were highly frequent during exposure.

### Alternative Explanations: Length of the Delay, Time of Exposure

The sleep groups had a longer delay between exposure and testing than the wake groups ([12 h, 24 h] versus [15 min, 15 min, 12 h] respectively). Also the exposure phase of the sleep groups always took place in the evening, while 2 out of 3 wake groups had the exposure phase in the morning. Therefore, we tested whether the time of the delay and the time of exposure and testing interacted with the effect of grammaticality and ACS on endorsement using planned comparisons (contrast 2– contrast 4, **Table 3**.).

To examine whether the length of the delay could have been responsible for the observed differences between the groups in contrast 1, we compared the 15 min wake groups with the 12 h wake group in contrast 2. No significant interactions between delay length and any other factors were found (all P>0.063, **Table 6**). Also when we compared the two sleep groups (contrast 3), which had a different delay length (12 h versus 24 h), again no interactions between delay length and any other factors were significant (all P>0.20, **Table 6**.) In summary, it is unlikely that the effects of brain-state (wake, sleep) on the participant’s classification decisions were due to differences in length of the delay between exposure and testing.

We also tested whether a difference in exposure or testing time could have been driving the differences between the sleep and wake groups, by comparing the 15 min morning against the 15 min evening group (contrast 4). But also in this contrast we found no interactions between group**×**grammaticality (P = 0.61), group**×**ACS (P = 0.11), or group**×**grammaticality**×**ACS (P = 0.90; contrast 4, **Table 6**.).

### Performance on the Short-term Memory Task

Finally we compared performance on the short-term memory task (acquisition phase) to test whether the differences between the sleep and wake groups were not already present immediately after the acquisition phase. On average participants typed in 49% of the letters correct, (5–6 letters: 84%, 7–8 letters: 66%, 9–10 letters: 54%, 11–12 letters: 43%). There was no significant difference in performance during the short-term memory task between groups (F(4, 76) = 0.49, P = 0.74), or between the sleep and wake groups (contrast 1, **Table 3**), or any of the other planned comparisons (all P values >0.35).

## Discussion

Our findings demonstrate that the brain-state (wake or sleep) during an offline delay modulate the classification performance after artificial grammar learning. In particular, classification performance was enhanced after a delay containing sleep. A clear dissociation was seen in the route by which this benefit of sleep developed: the improvement after sleep was due to an enhanced ability to discriminate between Grammatical and Non-Grammatical sequences (grammaticality effect) and was not be explained by an increased ability to recognize highly frequent chunks and reject low-frequency chunks (effect of ACS). Notably, the effect of offline sleep time on grammar acquisition did not appear to depend on the length of the offline delay or the time of day of either exposure or classification.

Sleep did not increase the proportion of sequences with highly frequent chunks (High ACS) that was endorsed as Grammatical. Participants in all groups endorsed a higher proportion of High than Low ACS sequences, but sleep did not modulate this effect. This is interesting considering that it’s known that one of the routes modulating classification performance is becoming familiar with sequence chunks that were highly frequent during exposure [11], [12], [22]. Moreover, it has been shown repeatedly that one of the effects of sleep is to strengthen individual memories (e.g. [29], [30], [31], [32]). Hence, strengthening of the memory for highly frequent chunks could have represented one possible route through which sleep improves classification performance; however, we did not find evidence for this.

Instead, it appears that the classification performance was improved via the grammaticality route, since after sleep, the ability to discriminate between Grammatical and Non-Grammatical sequences increased significantly. It has been argued that the ability to correctly judge grammaticality status during classification arises from integration and abstraction of the grammatical information present in the collective Acquisition Set of sequences used during exposure to the grammar [7], [8], [11]. The effect of sleep on the grammaticality route suggests that sleep promotes such integrative processes. During sleep, high-level rule abstraction processes appear to take place in which memories of individual sequences are combined, resulting in abstract knowledge reaching beyond the previously encountered exemplars. This knowledge might be a partially veridical representation of the underlying grammar rules. However, this does not imply that participants have acquired full “knowledge” of the grammar rules, either implicit or explicit, in this study, since they still classify many sequences incorrectly. Over time and with further implicit exposure, one would expect, however, that this knowledge would effectively converge on a representation equivalent to the underlying grammar, as in for example natural language acquisition [13], [25], [33].

Since Reber [7], artificial grammar learning is one of the main paradigms to investigate implicit learning. It is typically assumed that the classification performance is based on implicit acquisition mechanisms because subjects are in general unable to provide sufficient reasons to motivate their classification decisions [9], [14], [16], [22], [25]. It has also been argued that learning based on ACS reflects explicit declarative memory mechanism involving the medial temporal lobe [23], [34], while implicit learning of grammaticality status independent of ACS reflects an implicit procedural learning mechanism involving the basal ganglia and the prefrontal cortex [22], [24]. Although we did not test for explicit knowledge directly in the current study, previous results obtained in very similar artificial grammar learning paradigms [13], [14], [25] suggests that classification performance is to great extent based on implicit acquisition mechanisms. If this is so, then the current results suggest that sleep also enhances implicitly learned material, and might therefore not be limited to consolidating explicitly learned material [35], [36]. As noted in the introduction, we use the terms “implicit” and “implicit learning” to mean a lack of meta-cognitive knowledge and in particular the absence of any stated use of explicit “problem solving” strategies [7], [8], [9]. We note that recent experimental findings suggest that partial subjective awareness of the structural knowledge can develop in some artificial grammar learning tasks [17], [18], [19] and that a detailed investigation of the effect of sleep on the development of partial awareness would be a topic for future studies.

Since brain activity during sleep was not measured directly, it cannot be excluded that an unidentified factor occurring during the night time portion of the diurnal cycle, co-occurring with but independent of sleep, contributed to the reported findings, although this seems implausible. Nonetheless, several studies report a correlation between the level of improvement on associative and relational memory tasks and the amount of (a specific stage of) sleep, suggesting an active role for sleep [37], [38], [39]. Both Rapid Eye Movement (REM) sleep [37], [40] and Non-REM (NREM) sleep [38], [39] have been associated with improvements in the reprocessing and optimization of the high-level information contained in the learned material.

Several studies have shown that different groups of brain regions are selectively sensitive to the grammaticality status and to the ACS level of sequences, respectively [22], [23], [24]. Reduced activation levels for High versus Low ACS items were shown in early visual regions and the medial temporal lobe, while the caudate nucleus appears to be more active for Grammatical versus Non-Grammatical items [22], [23], [24]. Moreover, Broca’s region is sensitive to the grammaticality status of items and is not sensitive to ACS [22], [24], [26]. Future research will have to elucidate whether increased activity in the brain regions underlying the grammaticality route can be observed during sleep and whether such activity predicts post-sleep improvement of rule abstraction performance.

Our findings stress the importance of alternating periods of exposure to a new grammar with periods of sleep for optimal grammar acquisition. More generally, a night of sleep can be beneficial after exposure to material that requires high-level rule abstraction.

## Supporting Information

Appendix S1 **Task instructions exposure phase.** A) Written instructions, handed out to participants prior to short-term memory task. B) On screen instructions, appeared on computer screen before the short-term memory task started. Instructions were translated from Dutch.
(DOCX)

Appendix S2 **Task instructions test phase.** A) Written instructions, handed out to participants prior to the classification task. B) On screen instructions, appeared on computer screen before the classification task started. Instructions were translated from Dutch.
(DOCX)
