# Supplementary material for: Sleep Promotes the Extraction of Grammatical Rules
Source: PLoS One. 2013 Jun 5;8(6):e65046. doi: 10.1371/journal.pone.0065046 (PMC3673983; doi:10.1371/journal.pone.0065046)
Supplement: Appendix S1 — Task instructions exposure phase. A) Written instructions, handed out to participants prior to short-term memory task. B) On screen instructions, appeared on computer screen before the short-term memory task started. Instructions were translated from Dutch. (DOCX) [file pone.0065046.s001.docx]

**Appendix S1 Task instructions exposure phase.** (translated from Dutch)

*A: written instructions*

**"Instructions: Short-term Memory Task**

You will perform a working memory task. You will see letter sequences presented letter by letter on the screen. During the presentation of the sequences, read every letter carefully and remember it. After the last letter of the sequence a dot will be presented. When the sequence is done, you have to type the letter sequence as well as possible on the keyboard. When you mistype a letter, you can use the SPACEBAR to erase the last letter. When you are satisfied with the sequence you typed in, you can start the next sequence by pressing ENTER. If you cannot remember the whole sequence exactly, try to just type the part that you do remember, or guess as well as you can, but *do not keep thinking too long* about it, and keep a good pace. Try to stay focused, and attempt every sequence, even if it is long or difficult. After one hundred sequences (you can see how far you are on the top of the screen), *end of block* will appear on the screen. When that happens, go to the hall quietly (without disturbing the person next to you)^[[1]](#footnote-1)^, where you will have a short break before the next block starts.

The block will take approximately 30 minutes."

*B: On screen instructions*

"Short time memory task:

Shortly, letter sequences will appear letter by letter or the screen. Read and remember the sequence concentrated. After the last letter is presented (which can be noticed by the dot after the letter), type in the whole sequence to your best ability on the keyboard. You can delete using the SPACEBAR, and continue to the next sequence using ENTER.

Type in each sequence to your best ability, guess some letter when needed, but don't think too long if you do not remember. Do keep on reading and remembering every sequence while concentrating, and type it in as well as you can. After the block, go to the hallway for a short break.

Start by pressing a key"

1. An experimenter was present in the hall; participants were not allowed to talk about the task. [↑](#footnote-ref-1)
