# Supplementary material for: Sleep Promotes the Extraction of Grammatical Rules
Source: PLoS One. 2013 Jun 5;8(6):e65046. doi: 10.1371/journal.pone.0065046 (PMC3673983; doi:10.1371/journal.pone.0065046)
Supplement: Appendix S2 — Task instructions test phase. A) Written instructions, handed out to participants prior to the classification task. B) On screen instructions, appeared on computer screen before the classification task started. Instructions were translated from Dutch. (DOCX) [file pone.0065046.s002.docx]

**Appendix S2 Task instructions test phase.** (translated from Dutch)

*A: written instructions*

"**Instructions: Judging grammaticality**

The sequences you saw during the short term memory task were generated according to a complex set of grammar rules. They were all grammatical. Because the system behind the rules is very complex, it is much too difficult to discover these underlying rules. There's no use in trying, because you have to consider the sequence *as an entity* before you can determine if the sequence is grammatical or not.

During the following task, you will see new letter sequences. You have to read each sequence carefully to be able to decide if these new sequences are grammatical or not. The best strategy is to use your gut feeling (guess based on your immediate feeling about the sequence) while you do take *the whole sequence* into account. Decide based on the first feeling that emerges inside about whether the whole letter sequence feels good or not, and don't try to think about it.

When the whole sequence is presented (the last letter has a dot behind it again), a * appears on the screen. Press the RIGHT SHIFT key when you think the sequence is grammatical, and the LEFT SHIFT key when you think the sequence was not grammatical. Don't think, press based on your immediate gut feeling, the program will not wait and continues automatically to the next sequence.

This task will take about 20 minutes."

*B: On screen instructions*

"INSTRUCTIONS CLASSIFICATION GRAMMATICALITY

- Press the RIGHT SHIFT when the sequence is GRAMMATICAL.

- Press the LEFT SHIFT when the sequence is NOT GRAMMATICAL.

- Take the WHOLE sequence into account.

- Trust your first gut feeling.

- Do NOT think about it, don't try to come up with the rules.

- Keep concentrating on every sequence.

- Start by pressing the SPACEBAR."
